# *Dasiphora parvifolia*: Pharmacognostic Standardization and Antimicrobial Potential

**DOI:** 10.3390/ph19091443

**Published:** 2026-09-11

**Authors:** Aigul Medeshova, Aizhan Essedova, Margarita Ishmuratova, Perizat Orazbayeva, Piotr Belniak, Sabira Sabyrova

**Affiliations:** 1 School of Pharmacy, Karaganda Medical University, Gogol 40, Karaganda 100000, Kazakhstan; medeshova@qmu.kz (A.M.); perizat_abay@mail.ru (P.O.); sabyrovas@qmu.kz (S.S.); 2 Research Park of Biotechnology and Eco-Monitoring, Buketov Karaganda National Research University, Universitetskaya Street, 28, Karaganda 100028, Kazakhstan; 3 Department of Applied and Social Pharmacy, Faculty of Pharmacy, Medical University of Lublin, 1 Chodźki Street, 20-093 Lublin, Poland; belniako@wp.pl

**Keywords:** *Dasiphora parvifolia*, pharmacognostic standardization, ultrasound-assisted extraction (UAE), phenolic compounds, high-performance liquid chromatography (HPLC), antimicrobial activity

## Abstract

**Background/Objectives**: *Dasiphora parvifolia,* a little-studied Central Asian shrub, lacks standardized pharmacognostic data. This study aimed to comprehensively characterize its above-ground organs, establish diagnostic criteria, comparatively evaluate extraction methods, profile phytochemicals, and evaluate antimicrobial activity. **Methods**: Morphological, morphometric, and anatomical analyses were performed using light microscopy (ANOVA, *p* ≤ 0.05). Physicochemical parameters were determined gravimetrically. Percolation and ultrasonic-assisted extraction (UAE) were compared using varying ethanol concentrations, ratios, and maceration times. Phenolics were identified by HPLC-UV and confirmed by ESI-MS. **Results**: Diagnostic features included leaflet size (6.91 × 1.14 mm), stem length (17.39 cm), and flower diameter (6.92 mm). Microscopy revealed anomocytic stomata and multicellular trichomes (120 µm). Quality parameters (moisture 2.61%, total ash 5.55%, extractives 42.36%). UAE with 70% ethanol (1:20, 24 h pre-maceration) gave 7.53% total phenolics in the dry extract and 7.68% for percolation, corresponding to 20.81 and 28.72 mg/g of raw material, respectively. Among the analyzed extracts, the highest concentrations were detected for gallic acid (3.61%), chlorogenic acid (2.94%), cynaroside (2.28%), and hyperoside (1.82%). Total polyphenols, flavonoids, tannins, β-carotene, and α-tocopherol were 3.70%, 1.36%, 2.67%, 330.08 mg/kg, and 138.0 mg%, respectively. DPPE70 showed the highest activity against *P. aeruginosa* (17.0 mm), whereas DPUE70-24 showed the highest activity against *S. aureus* (22.0 mm). **Conclusions**: *D. parvifolia* can be considered a relevant source of phenolic compounds. The high levels of gallic and chlorogenic acids, together with hyperoside and cynaroside, indicate a substantial phenolic profile and may contribute to the antioxidant and antimicrobial activities of the extracts.

## 1. Introduction

According to the World Health Organization, more than 21,000 plant species are used for medicinal purposes worldwide, and approximately 25% of modern pharmaceutical drugs contain active substances of plant origin or have been developed on the basis of natural molecular prototypes [1,2,3]. These data confirm the continuing importance of natural compounds for the discovery and development of new pharmaceutical substances.

Of particular interest are plants that contain chemically diverse complexes of phenolic acids, flavonoids, tannins, terpenoids, and other secondary metabolites, for which antioxidant, anti-inflammatory, and antimicrobial properties have been described [1,2,3]. However, the detection of biologically active compounds is insufficient: the introduction of a new species into pharmaceutical practice requires its botanical identification, macro- and microscopic diagnostics, assessment of quality parameters, selection of a reproducible extraction technology, chemical profiling, and experimental evaluation of biological activity [4,5,6].

The choice of extraction method is of particular importance in the development of plant-based pharmaceutical substances, as the process parameters can significantly alter the qualitative and quantitative composition of the resulting phytocomplex. A previous study of plant raw materials from the Karaganda region demonstrated that percolation, vortex, microwave and ultrasonic extraction yield chemically distinct profiles [7]. The extract showed the strongest combined antioxidant and antimicrobial activity, while the ultrasonic extract contained 6.90% 5-hydroxymethylfurfural, suggesting that some components may have been transformed during extraction [7].

Thus, the choice of extraction method should be based not only on the maximum yield of extractive substances, but also on the preservation of biologically significant components, the reproducibility of the chemical profile, and the activity of the resulting extract. In the present study, percolation (a classical method with proven efficacy) and ultrasonic-assisted extraction (a promising industrial technique that significantly reduces extraction time) were selected for comparative evaluation.

Plants from arid and sub-arid regions are of particular interest as sources of new biologically active compounds. Central Kazakhstan, including the Karaganda region, is characterised by a sharply continental climate, insufficient moisture, intense insolation, and pronounced seasonal temperature fluctuations [8]. These factors may influence the secondary metabolism of plants and determine the geographical variability in the content of phenolic compounds, flavonoids, and other protective metabolites [4,5,6,7,8]. However, for each species and individual population, experimental verification is required, since the geographical origin of the raw material may affect its chemical composition, quality parameters, and the reproducibility of biological activity.

One such object is *Dasiphora parvifolia*—a shrub species of the family Rosaceae, distributed in Central Asia, including the territory of Kazakhstan. The genus *Dasiphora* Raf. comprises about 8–9 shrub species, predominantly found in Asia, with the exception of *D. fruticosa*, which has a circumboreal range [9]. The systematic position of members of the genus has long remained debatable: they were either assigned to the genus *Potentilla* L. or placed into the separate genus *Pentaphylloides* Hill [10]. Consequently, the main synonyms of *Dasiphora parvifolia* are *Potentilla parvifolia* Fisch. ex Lehm., *Pentaphylloides parvifolia* (Fisch. ex Lehm.) Soják, *Potentilla fruticosa* var. *parvifolia* (Fisch. ex Lehm.) Wolf, and *Dasiphora fruticosa* var. *parvifolia* (Fisch. ex Lehm.) Kartesz [10,11,12].

Such taxonomic ambiguity has not only botanical but also pharmaceutical significance, as it may complicate the correct identification of plant material and the comparison of phytochemical data published under different species names.

Species of the genus *Dasiphora* have been traditionally used in the folk medicine of Mongolia, Tibet, and adjacent regions. The best-studied representative is *Dasiphora fruticosa* (L.) Rydb., in the above-ground organs of which flavonoids, phenolic acids, hydrolysable tannins, ellagic acid, and other phenolic compounds have been identified [13,14,15,16,17,18,19,20]. Antioxidant, anti-inflammatory, and antimicrobial activities have been established for extracts and individual compounds of this species [19,20]. These results confirm the pharmaceutical potential of the genus *Dasiphora*, but cannot be directly extrapolated to closely related species, since the qualitative composition, quantitative content of individual metabolites, and pharmacological properties of plants may differ substantially.

In contrast to *D. fruticosa*, *D. parvifolia* remains fragmentarily studied. Previously, 14 compounds were isolated from the above-ground parts of the plant, including two new chromone acylglucosides [19,21,22]. The total phenolic content in *D. parvifolia* reached 14.17 mg/g, which is higher than in two other *Potentilla* species [19]. The extract showed activity against Gram-positive bacteria and *Candida albicans* [21], and the chromatographic profile of the species differed from that of *D. fruticosa* [23]. Taken together, these data provide a rationale for considering *D. parvifolia* as a source of pharmaceutically relevant compounds [18,19,21,22,23,24].

A previous pharmacognostic study of the above-ground organs of *D. parvifolia* from the Karaganda region established the main diagnostic features at the macro- and microscopic levels [24]. However, quantitative morphometric characterisation, physicochemical quality parameters, comparative evaluation of extraction methods, extended phytochemical profiling, and biological activity assessment were not addressed.

To date, comprehensive standardisation of *D. parvifolia* raw material from populations of Central Kazakhstan within a unified pharmaceutical framework has not been reported. These gaps currently limit its consideration as standardised medicinal plant raw material.

The aim of the present study was to provide a scientific basis for the future pharmaceutical standardisation of the above-ground organs of *Dasiphora parvifolia* growing in Central Kazakhstan. To achieve this aim, the following objectives were addressed:

- Establishment of morphological and anatomical diagnostic features, supported by quantitative morphometric analysis;
- Determination of the physicochemical quality parameters of the raw material;
- Comparative evaluation of extraction methods, including percolation and ultrasound-assisted extraction;
- Extended phytochemical profiling by HPLC-UV/ESI-MS;
- Assessment of the antimicrobial activity of the obtained extracts.

The findings provide a basis for further evaluation of *D. parvifolia* as a standardisable medicinal plant raw material and a potential source of herbal pharmaceutical substances.

## 2. Results

### 2.1. Macroscopic and Morphometric Analysis of the Above-Ground Organs of Dasiphora parvifolia

In accordance with the aim of the present study, a macroscopic and quantitative morphometric investigation was conducted on the above-ground organs of *Dasiphora parvifolia*, considered as a promising medicinal plant raw material. The analysis included stems, compound leaves, individual leaflets, petioles, and flowers, with assessment of corolla and sepal dimensions.

**Figure 1:**
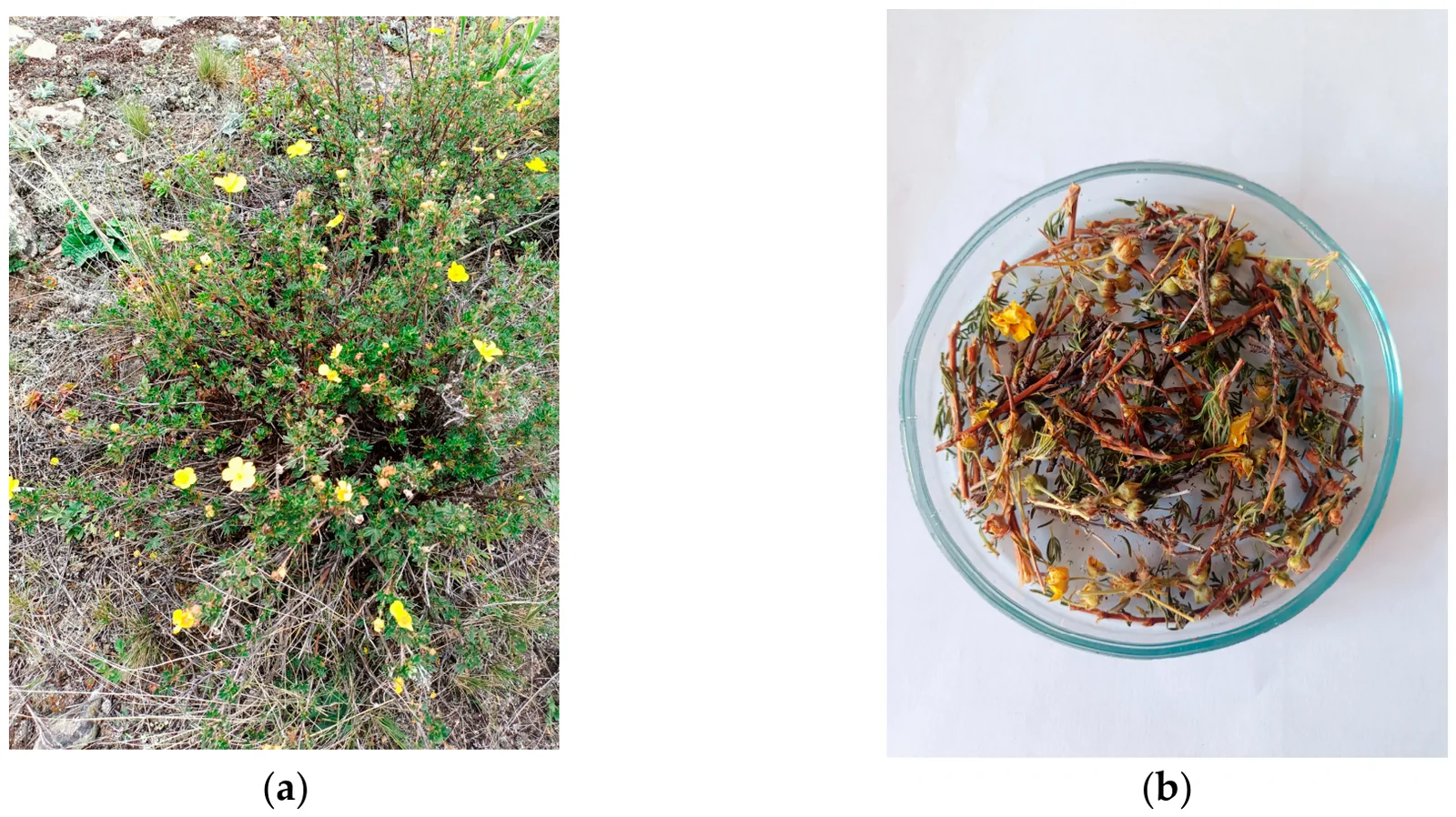
(**a**) Flowering plant of *Dasiphora parvifolia* in nature; (**b**) Crushed plant material of *Dasiphora parvifolia*.

The investigated raw material consisted of a mixture of thin, partially lignified stems, small greyish-green leaves with pronounced silky pubescence, and fragments of bright yellow flowers (Figure 1). The main qualitative morphological features of *D. parvifolia*, including leaf structure, pubescence characteristics, and flower morphology, have been previously described in our publication [24]. In the present study, these qualitative features were supplemented by quantitative morphometric evaluation, aimed at establishing objective diagnostic parameters relevant to the identification and standardisation of medicinal plant raw material (Table 1; see also Supplementary Table S1).

The obtained morphometric data complement the previously established qualitative characterisation of the species and form a quantitative basis for its pharmacognostic identification and standardisation. Together with diagnostic anatomical and microscopic features and physicochemical quality parameters, these measurements may be used to confirm the authenticity and assess the quality of the above-ground parts of *D. parvifolia* as a potential medicinal plant raw material.

Species of the genus *Dasiphora* may exhibit similar morphological features, which necessitates the establishment of a complex of diagnostic characteristics for the reliable identification of medicinal plant raw material. Accordingly, a comparative examination of the main morphological traits of the above-ground organs of *Dasiphora parvifolia, Dasiphora fruticosa*, and *Dasiphora phyllocalyx* was conducted. Comparative characteristics are presented in Table 2.

The comparative analysis demonstrated that identification of *D. parvifolia* should not be based on a single morphological character but requires evaluation of their totality. The most informative diagnostic features of the studied species are its low-growing subshrub life form, strongly branched partially lignified shoots, small compound leaves with predominantly 4–7 linear or linear-lanceolate leaflets, pronounced silky pubescence, especially on the lower leaf surface, as well as relatively small bright yellow flowers and the characteristic structure of the sepals.

The quantitative morphometric evaluation conducted in the present study further characterises *D. parvifolia* as a species with small vegetative and generative organs. The mean flower diameter was 6.92 ± 1.84 mm, compound leaf length and width were 9.31 ± 1.70 and 4.89 ± 0.93 mm, respectively, and individual leaflet length was 6.91 ± 0.64 mm. These parameters may serve as additional quantitative criteria for raw material identification.

In contrast to *D. fruticosa*, *D. parvifolia* is characterised by smaller flower size and narrower leaflets. From *D. phyllocalyx*, the studied species differs by the absence of markedly divided outer sepals and smaller flower dimensions. Furthermore, the pubescence characteristics and leaflet shape allow further differentiation of *D. parvifolia* from closely related species.

Thus, the combination of qualitative morphological and quantitative morphometric features may be used for pharmacognostic identification of intact and powdered *D. parvifolia* raw material.

### 2.2. Microscopical Investigation of the Above-Ground Organs of Dasiphora parvifolia

**Figure 2:**
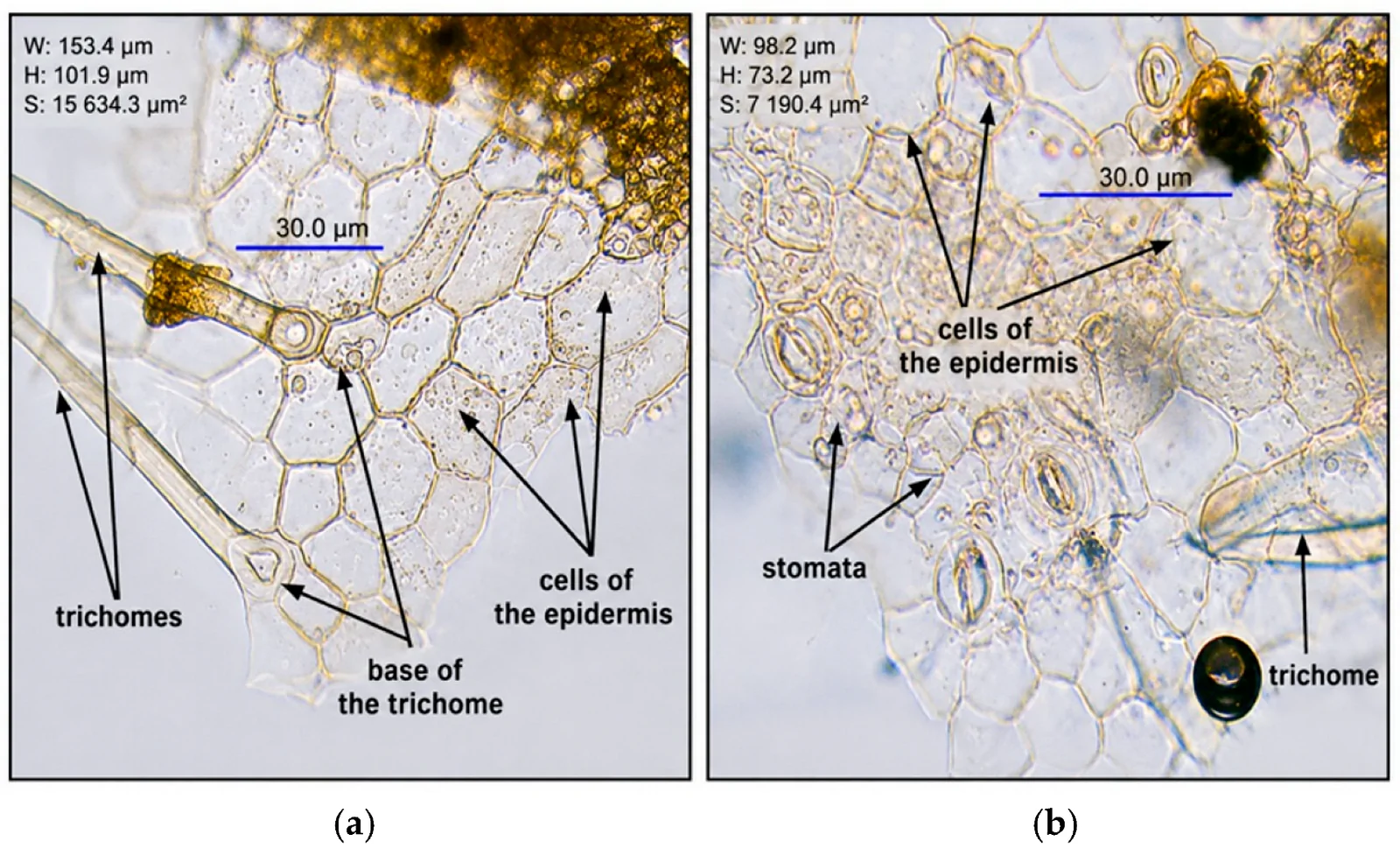
Surface preparation of the leaf epidermis of *Dasiphora parvifolia*: (**a**) upper epidermis (fragment); (**b**) lower epidermis with stomata and trichomes.

Microscopical examination revealed a complex of stable anatomical features of the above-ground organs of *D. parvifolia*. The leaf exhibited a dorsiventral structure and was covered by a uniseriate epidermis with a cuticle, more pronounced on the upper surface. Epidermal cells were predominantly polygonal in shape and tightly appressed to one another. Numerous stomata of the anomocytic type were observed on the lower leaf surface, while both surfaces of the leaf blade were covered with simple multicellular trichomes, more densely distributed on the abaxial side (Figure 2).

The leaf mesophyll was differentiated into palisade and spongy tissues. The palisade parenchyma consisted predominantly of two layers of elongated cells oriented perpendicularly to the leaf surface. The spongy parenchyma comprised 3–4 layers of loosely arranged cells with prominent intercellular spaces. The midvein contained a closed collateral vascular bundle, comprising xylem and phloem, surrounded by mechanical elements.

**Figure 3:**
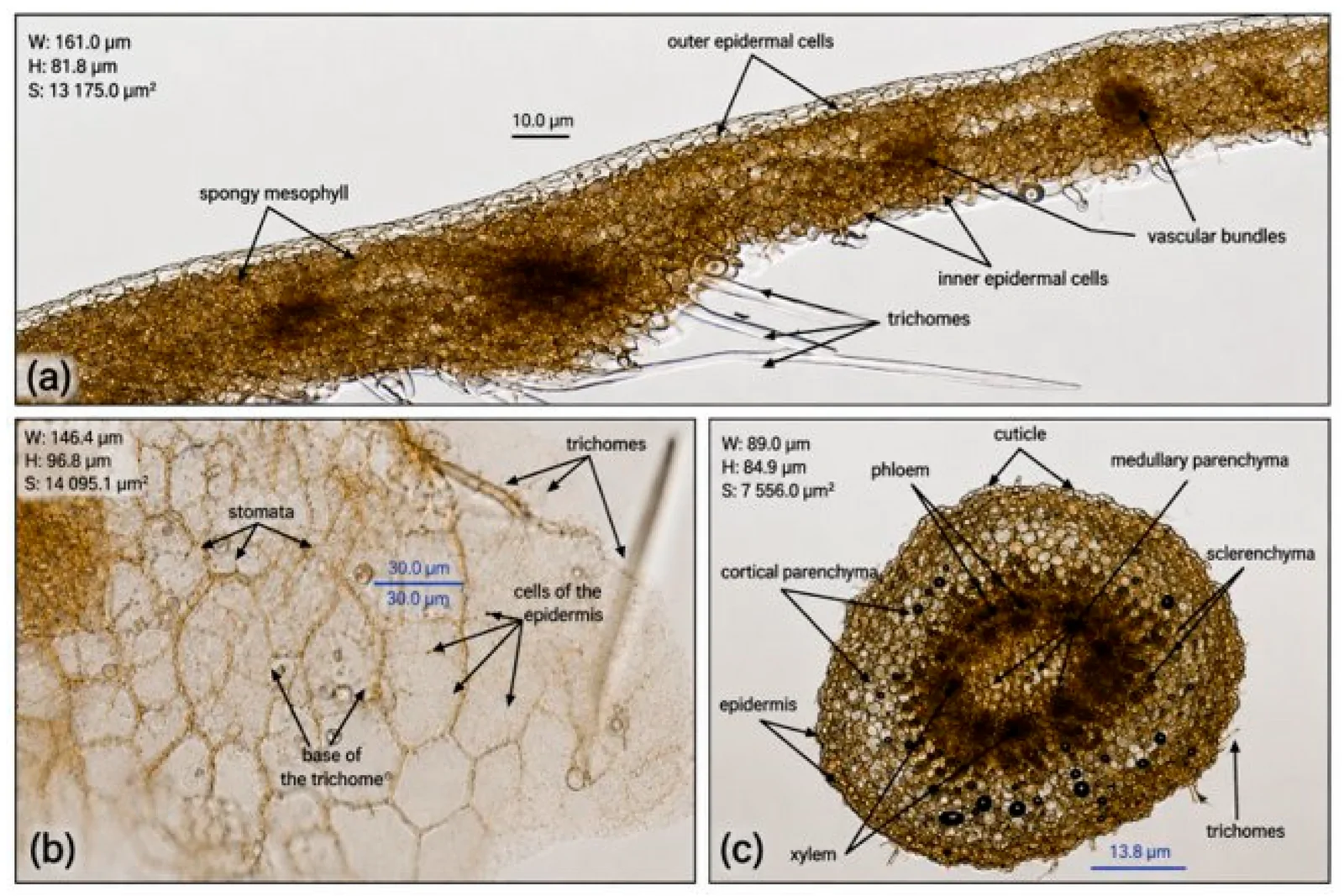
Anatomical structure of the organs of *Dasiphora parvifolia*: (**a**) transverse section of a sepal; (**b**) surface preparation of the sepal epidermis; (**c**) transverse section of a pedicel.

The sepals were characterised by differences between the outer and inner epidermis. The outer epidermis consisted of small rounded rectangular cells with sparse simple trichomes, whereas the inner epidermis was composed of larger rounded or oval cells. The sepal mesophyll was undifferentiated and included a multilayered spongy tissue (Figure 3a,b).

In the corolla, tightly appressed epidermal cells with a thin cuticle and yellow pigment inclusions were observed; stomata and trichomes were absent.

The stem in transverse section was rounded in outline and covered by exfoliating periderm. The vascular system was characterised by a non-fascicular type of organisation: the xylem formed a continuous ring divided by radial parenchymatous rays, while the phloem was arranged as a thin layer external to the cambial zone. The central part of the stem was represented by loose pith parenchyma. A similar anatomical organisation was noted for the pedicel; however, an additional multilayered collenchyma was observed beneath the epidermis (Figure 3c).

Quantitative assessment of the anatomical structures showed that the mean stomatal length and width were 27.0 ± 0.6 and 15.0 ± 0.6 µm, respectively, while the mean trichome length and mesophyll thickness were 120.0 ± 6.0 and 177.5 ± 4.5 µm, respectively. The palisade and spongy mesophyll layers measured 60.0 ± 6.0 and 107.5 ± 6.5 µm, respectively. The mean diameter of the vascular bundles was 37.5 ± 4.5 µm.

One-way ANOVA was used to compare the three collection sites for each anatomical parameter. No significant differences were detected among the collection sites for any of the measured parameters (F(2, 27) = 0.28–0.68, *p* > 0.05; see also Supplementary Table S2). Therefore, the data from the three sites were pooled for subsequent analysis.

### 2.3. Histochemical Localisation of Biologically Active Compounds

Histochemical analysis was performed to determine the distribution of major groups of secondary metabolites in the above-ground organs of *D. parvifolia*. Positive reactions for phenolic compounds and flavonoids were recorded in all studied organs; however, the intensity and tissue localisation of staining varied. Reactions indicative of phenolic compounds were predominantly observed in the leaf mesophyll, vascular tissues, bark, and xylem elements. Positive histochemical reactions consistent with flavonoids were observed in the leaf epidermis and mesophyll, stem cortical parenchyma, and vascular tissues of the pedicel. Weak staining with Dragendorff’s reagent suggested the possible presence of trace amounts of alkaloids, mainly in the mesophyll and vascular regions. Essential oils, sesquiterpene lactones, polysaccharides, and starch were not detected by the employed reagents.

It should be noted that histochemical reactions with FeCl_3_ and Dragendorff’s reagent are not entirely specific: FeCl_3_ detects a broad range of phenolic compounds, while Dragendorff’s reagent provides only presumptive evidence for alkaloids.

The results of the histochemical analysis of individual metabolite groups are presented in Table 3.

The obtained pattern of metabolite distribution is consistent with the anatomical structure of the studied organs. The intense reaction for phenolic compounds in the leaf mesophyll and vascular tissues may be related to their involvement in the antioxidant defence of photosynthetic tissues and the plant’s response to external stress factors. The accumulation of phenolic components in the bark and xylem may also be significant for structural and chemical protection of perennial shoots.

The most reproducible histochemical feature of *D. parvifolia* was the combined detection of phenolic compounds and flavonoids in leaves, stems, corollas, and pedicels. However, the detection of alkaloids was weak; therefore, this feature should be interpreted as preliminary and confirmed by specific chromatographic methods. The results of histochemical analysis complement the macro- and microscopic features and may be used in the comprehensive characterisation and development of identification criteria for plant raw material.

**Figure 4:**
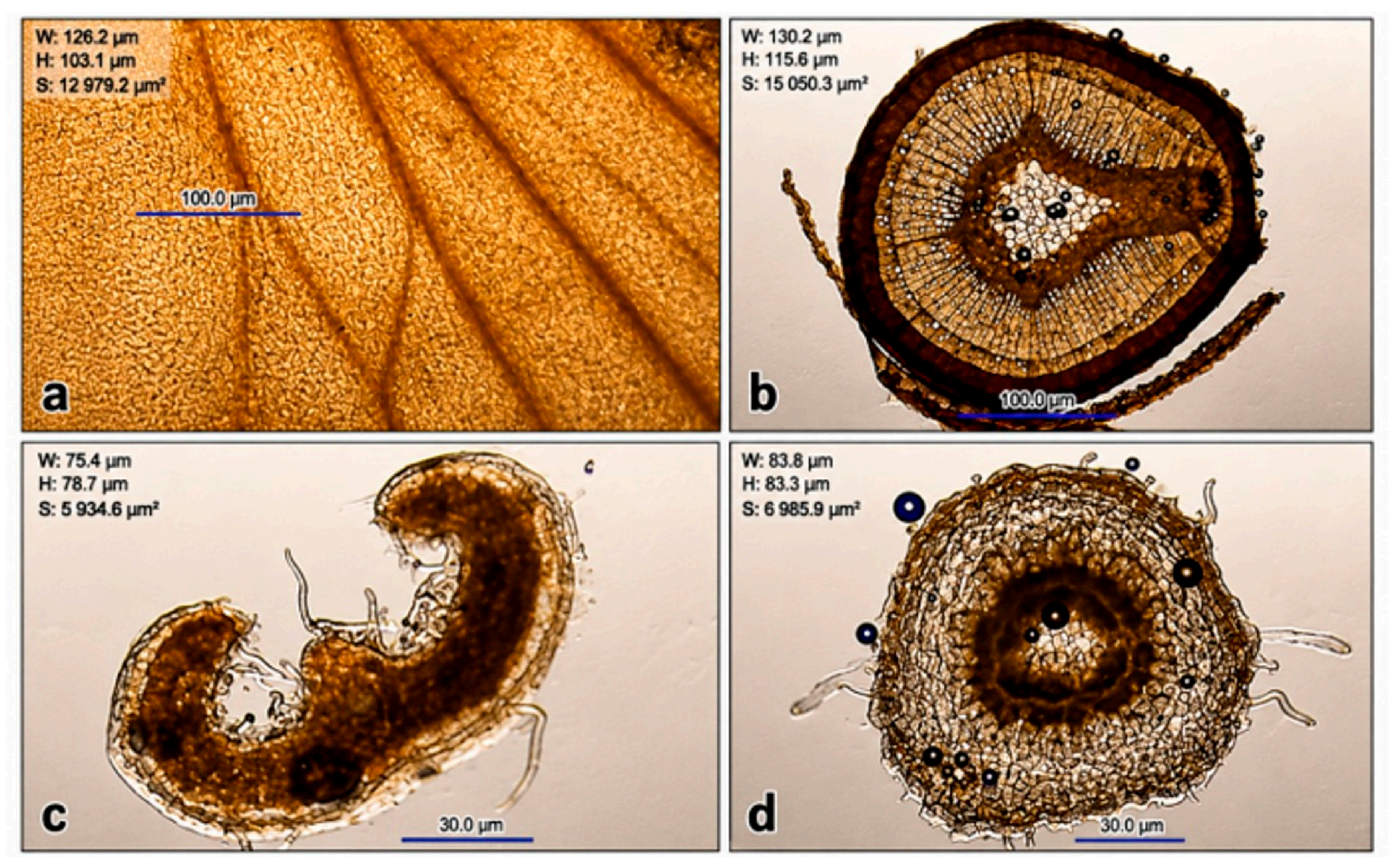
Results of histochemical reactions with 1% alcoholic FeCl_3_ solution. (**a**) Corolla; (**b**) Stem cross-section; (**c**) Leaf cross-section; (**d**) Peduncle cross-section.

Histochemical analysis of the above-ground organs of *Dasiphora parvifolia* revealed positive reactions consistent with flavonoids in all studied samples (Figure 4). In transverse sections of leaves, flavonoids were localised in the epidermis and mesophyll, with the most intense colouration observed in the vascular tissues. In the corolla, flavonoids were mainly localised in the epidermis. In transverse sections of the stem, flavonoids were detected in the bark and cortical parenchyma, while weaker staining was observed in the xylem vessels and pith parenchyma. In the pedicel, flavonoids were localised in the cortical parenchyma and vascular bundles.

Such localisation is consistent with a potential role of flavonoids in antioxidant defence and transport processes.

**Figure 5:**
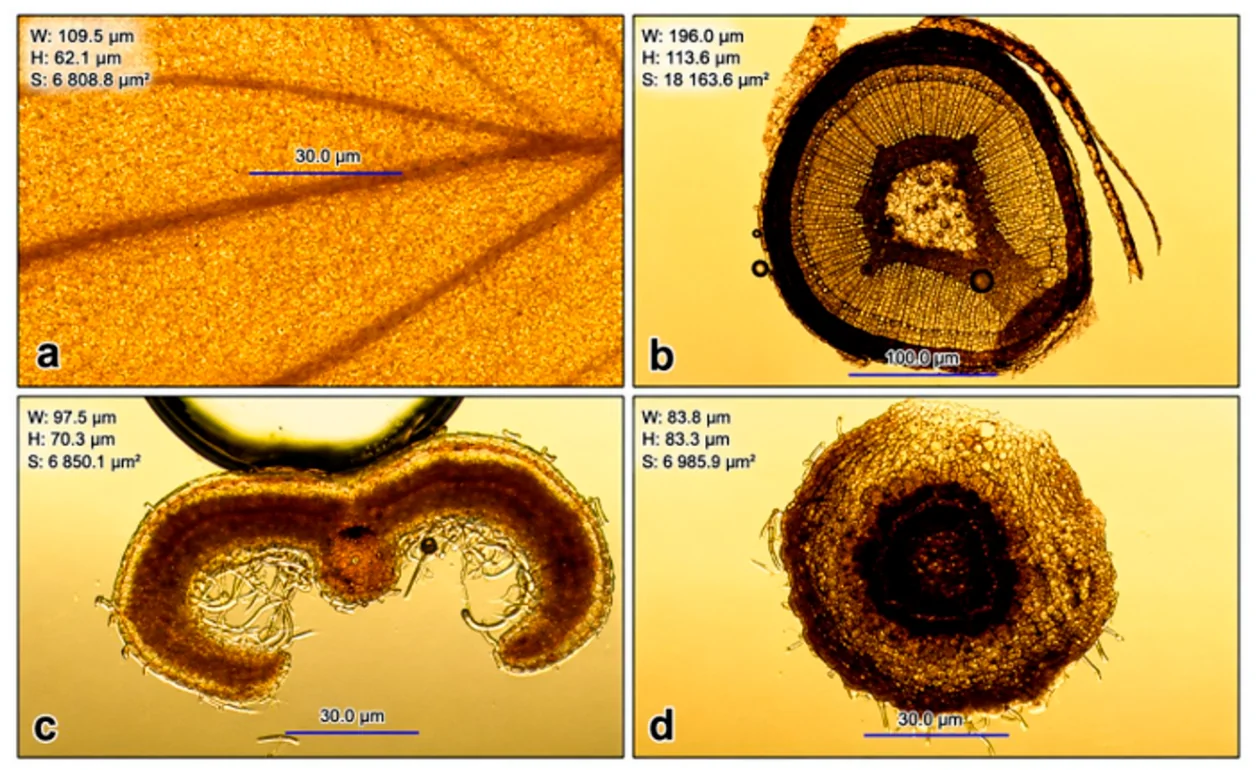
Results of histochemical reactions with 10% alcoholic solution of K_2_Cr_2_O_7_. (**a**) Corolla; (**b**) Stem cross-section; (**c**) Leaf cross-section; (**d**) Peduncle cross-section.

Reactions indicative of phenolic compounds were also observed in all studied preparations (Figure 5). In transverse sections of leaves, intense colouration was observed in the mesophyll tissues and vascular bundles, whereas the epidermal tissues stained less intensely. In corolla preparations, the veins corresponding to the vascular bundles were stained, while the epidermal cells exhibited weaker colouration. In transverse sections of stems, phenolic compounds were localised in the bark and xylem tissues. In transverse sections of the pedicel, phenolic compounds were present in the vascular bundles and pith parenchyma. Phenolic compounds in *D. Parvifoli* are predominantly concentrated in the vascular tissues and mesophyll, suggesting that they play a role in structural defence mechanisms.

**Figure 6:**
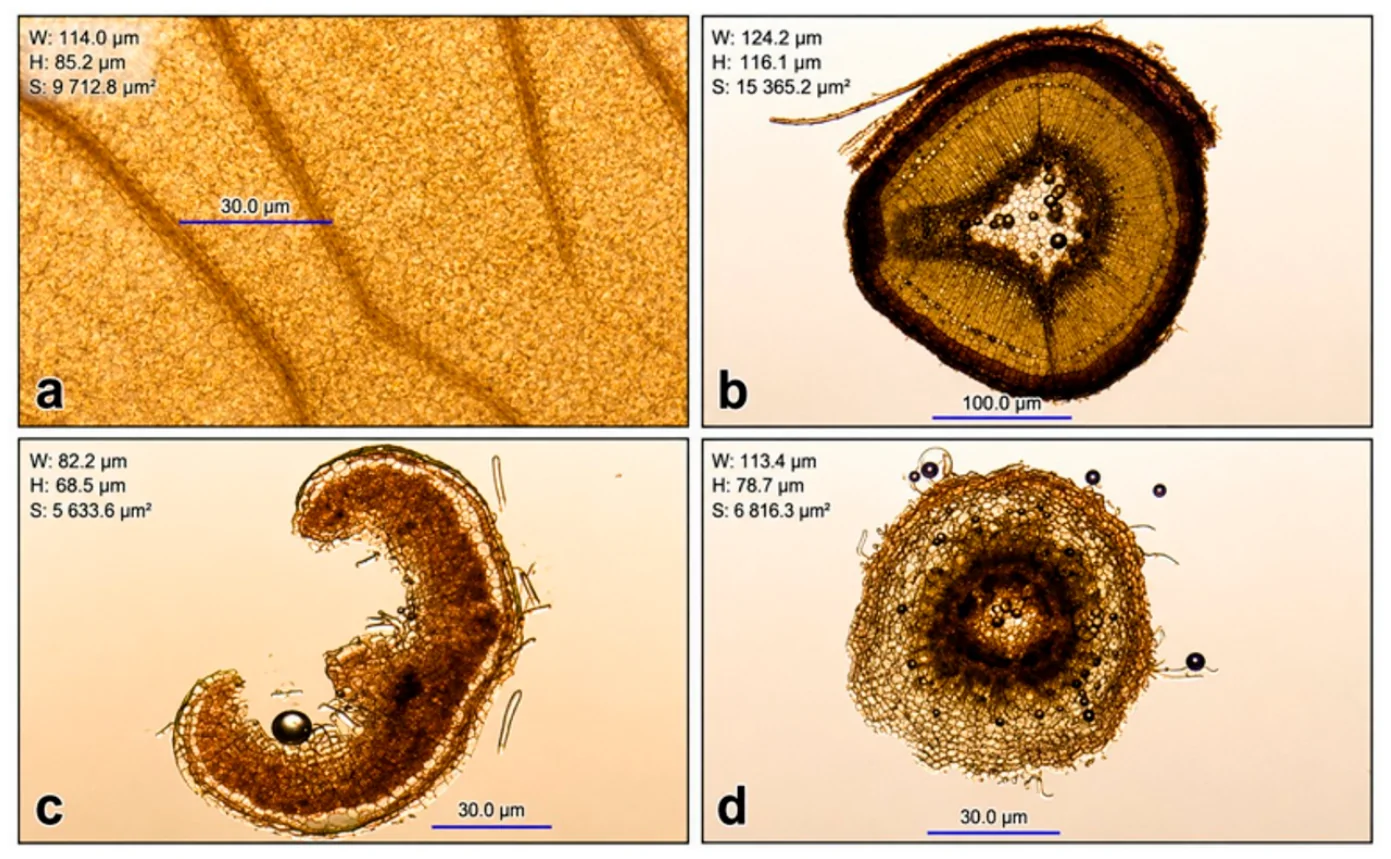
Results of histochemical reactions with Dragendorff’s reagent. (**a**) Corolla; (**b**) Stem cross-section; (**c**) Leaf cross-section; (**d**) Peduncle cross-section.

When stained with Dragendorff’s reagent, colouration indicative of the presence of trace amounts of alkaloids was observed. Characteristic staining was detected in the mesophyll cells of leaf transverse sections, along the vascular bundles of the corolla, in the bark and vascular region of stem transverse sections, as well as along the vascular bundles of pedicel transverse sections (Figure 6).

### 2.4. Quality Assessment of Raw Material and Characterisation of Major Groups of Biologically Active Compounds

Quality assessment of the above-ground parts of *D. parvifolia* included the determination of parameters characterising the condition and purity of the plant raw material, its mineral residue, and the capacity of various solvents to extract the complex of soluble components. The loss on drying was 2.61 ± 0.02%, indicating a low residual moisture content in the studied samples. The total ash content was 5.55 ± 0.04%, ash insoluble in 10% hydrochloric acid solution was 0.46 ± 0.07%, and extraneous impurities accounted for 0.21 ± 0.007% (Table 4).

The obtained values may serve as preliminary experimental reference points for the subsequent development of a quality specification for *D. parvifolia* raw material.

The content of extractive substances was substantially dependent on ethanol concentration. When water was used as the extractant, this parameter was 36.09 ± 0.24%, whereas increasing ethanol concentration from 30% to 70% was accompanied by a progressive increase in extractive yield from 37.61 ± 0.20% to 42.36 ± 0.50%. Further increase in ethanol concentration to 90% resulted in a decrease in the parameter to 31.32 ± 0.21%. Thus, maximum extractability was achieved using 70% ethanol.

This observed dependence indicates that the major portion of soluble components of the raw material is represented by compounds requiring a moderately polar hydroalcoholic system for their extraction.

For extended chemical characterisation of the above-ground parts of *Dasiphora parvifolia*, the content of hydrophilic and lipophilic components capable of contributing to the antioxidant properties of the plant raw material was determined. The obtained results are presented in Table 5.

In the hydrophilic fraction, total polyphenols, flavonoids, tannins, ascorbic acid, and water-soluble antioxidants were quantitatively determined. The total polyphenol content was 3.70 ± 0.04 mg-equiv gallic acid/g of absolutely dry raw material, and total flavonoid content was 1.36 ± 0.01 mg-equiv rutin/g. The amount of tannins reached 2.67 ± 0.03%, ascorbic acid content was 11.2 ± 0.10 mg%, and water-soluble antioxidants amounted to 1.81 ± 0.0096 mg-equiv quercetin/g.

In the lipophilic fraction, the content of β-carotene was 330.08 ± 2.65 mg/kg and α-tocopherol was 138.0 ± 0.8 mg%. The total content of fat-soluble antioxidants was 0.87 ± 0.0014 mg-equiv quercetin/g.

Thus, the above-ground parts of *D. parvifolia* contain a complex of compounds of varying polarity, including phenolic substances, flavonoids, tannins, ascorbic acid, carotenoids, and tocopherols.

### 2.5. Comparative Evaluation of Extraction Conditions and Phenolic Profiles of Extracts

Following pharmacognostic characterisation and assessment of the overall chemical composition of the raw material, a comparative evaluation of various methods for obtaining *Dasiphora parvifolia* extracts was conducted. The study employed extracts obtained by percolation and ultrasonic-assisted extraction using water and hydroalcoholic solutions of varying concentrations. The conditions for obtaining the investigated extracts are presented in Table 6.

For comparative evaluation of the phytochemical composition, all obtained extracts were analysed by high-performance liquid chromatography with ultraviolet detection. Identification of the major components was confirmed by HPLC-ESI-MS. The main chromatographic parameters are presented in Table 7.

As a result of the analysis, A total of eleven phenolic compounds were identified across all investigated extracts, including gallic acid, chlorogenic acid, caffeic acid, catechin, epicatechin, rutin, hyperoside, quercetin, quercetin-3-glucoside, cynaroside, and luteolin. The estimated content of individual components is presented in Table 8 (see Supplementary Materials, Figure S1 and Tables S3–S5).

Comparison of different extraction methods revealed that the composition and estimated content of phenolic compounds were substantially dependent on both the solvent used and the extraction technique. The highest total content of quantitatively determined phenolic compounds was recorded in the DPPE70 extract, obtained by percolation with 70% ethanol (7.79%). A practically comparable result was achieved with ultrasonic-assisted extraction using 70% ethanol following pre-maceration of the raw material (DPUE70-24), where the total phenolic content was 7.52%.

Analysis of individual compounds revealed that the maximum concentration of gallic acid was observed in the aqueous ultrasonic extract DPUEW5 (3.61%), whereas the highest chlorogenic acid content was determined in the DPUE70-24 extract (2.94%). Extracts obtained with 70% ethanol were also characterised by higher contents of hyperoside, rutin, quercetin, and cynaroside compared to extracts prepared with 90% ethanol.

Of particular interest is the effect of pre-maceration of the plant raw material. When using 70% ethanol, the total phenolic content increased from 4.85% to 7.52%, whereas the application of a similar approach with 90% ethanol was accompanied by a decrease in this parameter from 2.35% to 0.78%. The obtained results indicate that the efficiency of phenolic compound extraction is determined not only by solvent polarity but also by the degree of preliminary hydration of the plant tissue.

To enable a valid comparison of extraction efficiency across different methods and material-to-solvent ratios, the total identified phenolic content was recalculated from the percentage of the dry extract to mg per gram of the original dry raw material.

The recalculation was performed using the formula:

(1) $$X_{\mathrm{raw}}\left(mg/g\right)=\frac{C_{\mathrm{extract}}\left(\%\right)\times m_{\mathrm{dry}\;\mathrm{extract}}\left(g\right)}{10.0\;g}\times 1000$$

where $C_{\mathrm{extract}}$ is the total identified phenolic content in the dry extract (%) and $m_{\mathrm{dry}\;\mathrm{extract}}$ is the mass of the dry extract obtained from 10.0 g of raw material after correction for moisture content (22%).

The 22.0 ± 1.5% correction factor used for recalculation corresponds to the average moisture content of the thick extracts, as determined gravimetrically.

After recalculation (Table 9), the highest phenolic yield was observed for the percolation extract DPPE70 (28.72 mg/g), followed by the ultrasound-assisted extract with 24 h pre-soaking DPUE70-24 (20.81 mg/g). The aqueous extract DPUEW5 showed the lowest yield (3.64 mg/g).

### 2.6. Preliminary Antimicrobial Activity of the Extracts

Preliminary screening of the antimicrobial activity of *Dasiphora parvifolia* extracts by the agar diffusion method revealed differences in the susceptibility of the tested microorganisms depending on the extraction method (Table 10). The most pronounced activity was recorded against *Staphylococcus aureus*. The maximum growth inhibition zone diameter was demonstrated by the DPUE70-24 extract (22 ± 0.78 mm), whereas for DPPE70 it was 13 ± 0.50 mm.

Against *Pseudomonas aeruginosa*, the DPPE70 extract exhibited the highest activity (17 ± 0.40 mm), with the DPUE70-24 extract showing somewhat lower activity (15 ± 0.10 mm).

For *Bacillus subtilis*, all tested extracts exhibited moderate activity (6–8 mm), whereas against *Escherichia coli* and *Candida albicans*, the inhibition zones did not exceed the disc diameter (6 mm), indicating the absence of pronounced antimicrobial effect.

The activity of the tested extracts was inferior to the corresponding positive controls (cefoxitin, ampicillin, and nystatin); however, 70% ethanol, used as a solvent control, exhibited no inhibitory effect, confirming that the observed activity is attributable to biologically active compounds of plant origin.

These findings provide preliminary evidence of the antimicrobial activity of *D. parvifolia* extracts and support further investigation of their antimicrobial potential.

## 3. Discussion

The present study represents a comprehensive pharmacognostic investigation of *Dasiphora parvifolia* growing in Central Kazakhstan, integrating morphological, anatomical, histochemical, physicochemical, and phytochemical characterisation of the medicinal plant raw material. In contrast to most previously published works, which have focused predominantly on the investigation of individual groups of biologically active compounds or the evaluation of the biological activity of extracts, the present study is oriented towards establishing a scientific basis for the standardisation of the raw material and its further use in the development of phytopharmaceuticals.

### 3.1. Morphological and Anatomical Diagnostic Features

The obtained results demonstrated that *D. parvifolia* is characterised by a stable complex of diagnostic features enabling reliable differentiation of this species from closely related representatives of the genus *Dasiphora*. The most informative features are the small plant size (15–80 cm), linear or linear-lanceolate leaflet shape, the presence of 4–7 leaflets, small flowers with a diameter of 6.92 ± 1.84 mm, and a hypanthium with appressed silky pubescence. Collectively, these features are fully consistent with current botanical descriptions of the species and substantially expand the existing pharmacognostic knowledge, since previously most publications have focused primarily on the chemical composition of plants rather than on their diagnostic characteristics [18,19,20,21,22,23].

Of additional diagnostic value are the revealed anatomical features of leaves and stems. The dorsiventral structure of the leaf blade, the anomocytic stomatal apparatus, and the well-developed trichome cover are stable microscopic features that may be used in the development of pharmacopoeial requirements for raw material authenticity. Quantitative assessment of anatomical structures demonstrated that mean stomatal length was 27.0 ± 0.6 µm, width 15.0 ± 0.6 µm, trichome length 120.0 ± 6.0 µm, and mesophyll thickness 177.5 ± 4.5 µm. No statistically significant differences were found between samples from the three collection sites (*p* > 0.05), indicating the relative stability of the main anatomical characteristics of the species.

### 3.2. Histochemical Distribution of Secondary Metabolites

Histochemical investigations confirmed the pronounced tissue specialisation of secondary metabolites. The predominant localisation of flavonoids in the epidermis and mesophyll corresponds to their known photoprotective function, whereas the accumulation of tannins near vascular tissues reflects their involvement in mechanical protection and cell wall formation. Such distribution of phenolic compounds is characteristic of most representatives of the genus *Potentilla* and is considered one of the features of their adaptation to adverse growing conditions [26,27].

The absence of staining for essential oils and starch suggests that the leading role in the formation of the chemical profile of *D. parvifolia* belongs to compounds of phenolic nature, primarily flavonoids and hydrolysable tannins. The obtained results are in good agreement with current concepts of the phytochemistry of the genus *Potentilla*, where these classes of compounds determine the major part of the pharmacological activity [26,28].

### 3.3. Physicochemical Parameters and Quality of the Medicinal Raw Material

The investigated raw material was characterised by low moisture content (2.61%), total ash (5.55%), ash insoluble in 10% HCl (0.46%), and extraneous impurities (0.21%), indicating a high degree of purity of the plant material and minimal mineral contamination. Since no official pharmacopoeial monograph exists for *D. parvifolia*, the obtained parameters should be considered as reference values for the subsequent development of regulatory documentation.

The regular increase in extractive yield from 36.09% with aqueous extraction to 42.36% with 70% ethanol, followed by a decrease to 31.32% with 90% ethanol, reflects the changes in solubility of phenolic compounds of varying polarity. A similar dependence has been repeatedly described for phenol-containing medicinal raw materials, where hydroalcoholic systems of medium polarity provide the most complete extraction of flavonoids, phenolic acids, and hydrolysable tannins [26,27,29,30]. Consequently, the use of 70% ethanol is the most rational choice for obtaining standardised extracts of *D. parvifolia*.

### 3.4. Phytochemical Profile and Its Significance for Standardisation

The findings of this study confirm that *D. parvifolia* is a rich source of phenolic compounds. According to the data of Wang et al. (2013), six major phenolic compounds—(+)-catechin, caffeic acid, ferulic acid, hyperoside, rutin, and ellagic acid—were identified in the leaves of this species, with the total content of these compounds reaching 14.17 mg/g, which exceeds the corresponding values for *D. fruticosa* (10.01 mg/g) and *D. glabra* (7.01 mg/g) [19]. Moreover, the chromatographic profile of *D. parvifolia* includes 15 peaks, whereas only 11 common peaks were detected for the compared species, indicating a more complex phenolic composition of this species.

Of particular interest is hyperoside, which is the dominant individual compound in both *D. parvifolia* (2.68 mg/g) and *D. fruticosa* (8.86 mg/g) [19]. Despite the lower hyperoside content, it is *D. parvifolia* that is characterised by the maximum sum of identified phenols, suggesting that it is not a single compound but the totality of the phenolic profile that should be considered the most informative criterion for standardisation. Accordingly, HPLC profiling, together with the determination of hyperoside and the total content of the six target phenolic compounds, may be considered as suitable quality-control parameters for the standardisation of *D. parvifolia* medicinal raw material. Such an approach is consistent with the general principles of phytopharmaceutical standardisation, which emphasise comprehensive chemical characterisation rather than reliance on a single marker compound [26,27,28,29,30].

When the HPLC results were recalculated as absolute yields per gram of dry raw material, percolation with 70% ethanol (DPPE70) produced the highest phenolic yield (28.72 mg/g). The UAE procedure preceded by 24 h of maceration (DPUE70-24) yielded 20.81 mg/g, corresponding to approximately 73% of the percolation yield. At the same time, the total processing time for DPUE70-24 was approximately 25.5 h, including the 24 h pre-maceration period and 1.5 h of active sonication, compared with 48 h for the percolation procedure. Thus, although percolation provided a higher absolute phenolic yield, UAE with pre-maceration achieved a substantial proportion of this yield within a shorter overall processing period. The aqueous extract (DPUEW5) showed the lowest phenolic yield per gram of raw material, which was mainly attributable to its comparatively low dry extract yield.

Taken together, these findings indicate that UAE preceded by 24 h of pre-maceration may represent a feasible laboratory-scale alternative to conventional percolation when both extraction yield and processing time are considered.

### 3.5. Biological Activity of the Phenolic Complex

The high content of total polyphenols (3.70%), flavonoids (1.36%), and tannins (2.67%) in the investigated raw material confirms the leading role of phenolic compounds in the formation of its biological potential. It is well known that the antioxidant activity of representatives of the genus *Potentilla* correlates closely with the content of phenolic compounds [19,26].

Wang et al. (2013) conducted a comparative evaluation of the antioxidant activity of three *Potentilla* species, including *P. parvifolia* [19]. According to their data, *P. fruticosa* exhibited the highest antioxidant activity in all three assays (DPPH IC_50_—16.87 µg/mL; ABTS—2763.48 µmol TE/g; FRAP—1398.70 µmol TE/g). *P. parvifolia* also demonstrated significant activity: DPPH IC_50_—23.87 µg/mL, ABTS—2140.22 µmol TE/g, FRAP—1291.76 µmol TE/g [18]. Notably, *P. parvifolia* was characterised by the lowest total phenolic content (34.22 mmol GAE/100 g) compared to *P. fruticosa* (84.93 mmol GAE/100 g), but exhibited high reducing capacity in the FRAP assay, which may be attributed to the qualitative composition of the phenolic complex, including the presence of specific compounds such as chromone derivatives [19,20,21,22,23]. In the present study, the total polyphenol content in *D. parvifolia* raw material was 3.70%, and flavonoids 1.36%, which is consistent with literature data and confirms the significant contribution of phenolic compounds to the formation of biological activity.

Equally important is the antimicrobial potential of the species. According to literature data, *P. parvifolia* inhibited all tested microorganisms with an MIC range of 12.5–50 mg/mL, with the exception of *E. coli* [19]. In contrast, *P. fruticosa* demonstrated higher activity against certain sensitive strains, including *Candida albicans* (MIC 0.78 mg/mL) and *Pseudomonas aeruginosa* (MIC 6.25 mg/mL), but showed no activity against *Escherichia coli* and *Klebsiella pneumoniae* [19]. Thus, *D. parvifolia* is characterised by a broader spectrum of antimicrobial action, whereas *D. fruticosa* exhibits more pronounced activity against individual microorganisms. These differences are likely due to the qualitative composition of the phenolic complex and the presence of various minor components that may exert synergistic effects. Of particular interest is the high antifungal activity of *P. parvifolia*: according to Wang et al. (2013), this species showed the strongest inhibition of the phytopathogenic fungi *Alternaria alternata*, *Glomerella cingnlata*, *Physalospora piricola*, and *Venturia pyrina* with EC_50_ values of 25.71–47.02 mg/mL, which the authors attributed to the highest content of identified phenolic compounds and the greatest number of chromatographic peaks [19].

In the present study, screening by the agar diffusion method demonstrated that the DPUE70-24 extract exhibited maximum activity against Staphylococcus aureus (inhibition zone 22.0 mm), whereas DPPE70 showed the highest activity against Pseudomonas aeruginosa (17.0 mm). No activity was observed against *E. coli* and *C. albicans*, which is consistent with literature data for *D. parvifolia*, where the absence of activity against *E. coli* was also noted [19].

The observed differences between DPPE70 and DPUE70-24 may be hypothetically related to their distinct phenolic profiles: DPUE70-24 is enriched in chlorogenic acid (2.94%) and hyperoside (1.48%), whereas DPPE70 contains higher levels of cynaroside (2.28%) and chlorogenic acid (2.01%). However, this remains a hypothesis that requires confirmation through bioassay-guided fractionation and MIC determination.

A formal statistical correlation between individual phenolic compounds and antimicrobial activity was not performed in this preliminary screening study due to the limited number of active extracts (*n* = 2) and the semi-quantitative nature of disc-diffusion data.

### 3.6. Practical Significance of the Study and Prospects for Standardisation

*D. parvifolia* can be considered a potential source of medicinal plant raw material. Its morphological and anatomical characteristics enable reliable identification, while the established phenolic profile provides a basis for the development of standardisation approaches. Hyperoside, ellagic acid, and the total content of the six identified phenolic compounds may be considered candidate quality markers for the standardisation of *D. parvifolia* raw material. The use of multiple chemical markers reflects the multicomponent nature of phytopharmaceuticals and may provide a more comprehensive assessment of raw material quality than the determination of a single compound [26,27,28,29,30].

The present study represents a preliminary pharmacognostic characterisation based on plant material collected during a single vegetation period from three collection sites, with the samples subsequently pooled. Further studies involving samples collected over different vegetation periods and from a broader range of geographical locations are required to assess the variability and reproducibility of the established pharmacognostic and phytochemical characteristics.

## 4. Materials and Methods

### 4.1. Plant Material

**Figure 7:**
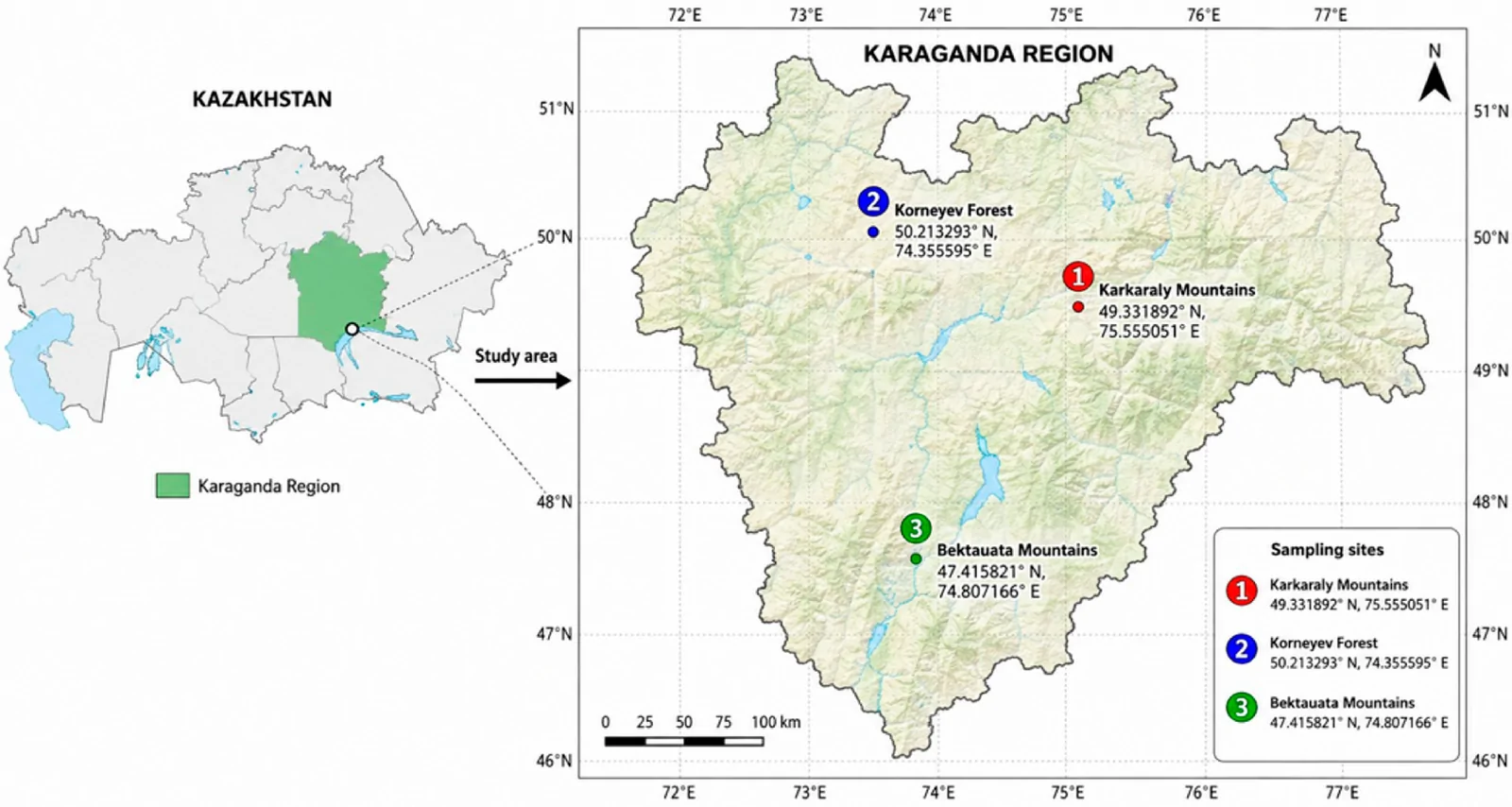
Map of locations where *Dasiphora parvifolia* has been collected in the Karaganda Region, Central Kazakhstan. Symbols: 1—Karkaraly Mountains; 2—Korneyev Forests; 3—Bektauata Mountains.

The above-ground organs of *Dasiphora parvifolia*, including stems, leaves, flowers (corollas and sepals), and pedicels, were used in this study. Plant material was collected during the mass flowering period, in mid-August 2025, from three geographically distinct locations in the Karaganda region, Central Kazakhstan (Figure 7):

1. Rocky hills of the Karkaraly Mountains—49.331892° N, 75.555051° E;
2. Korneev forests of the Bukhar-Zhyrau district—50.213293° N, 74.355595° E;
3. Bektauata mountain range of the Aktogay district—47.415821° N, 74.807166° E.

For morphometric and statistical analysis, 10 representative plants were selected from each site. For subsequent physicochemical, extraction, and chromatographic analyses, equal amounts of dried material from the three collection sites were pooled into a single composite sample. This was done to obtain a representative profile of the species in the region and to minimize the influence of local microclimatic differences. For morphometric and anatomical analyses, the three sites were analyzed separately.

The raw material was cleaned of mechanical and extraneous plant impurities, and then air-dried in the shade at room temperature in a well-ventilated room until constant mass was achieved. The dried samples were stored in paper bags in a dry, light-protected place.

Taxonomic identification of the plant material, as well as morphological, anatomical, and histochemical studies, were conducted at the Research Park of Biotechnology and Ecological Monitoring, Faculty of Biology and Geography, Karaganda National Research University named after Academician E. A. Buketov. Herbarium specimens were deposited in the herbarium of the Faculty of Biology and Geography under registration numbers QAR00576, QAR00621, and QAR07368.

### 4.2. Macroscopical Analysis

Macroscopical examination of the above-ground organs of *D. parvifolia* was performed using a Levenhuk DTX 50 digital microscope (Levenhuk Optics, Prague, Czech Republic) equipped with a 1.3 MP digital camera (Levenhuk Optics, Prague, Czech Republic).

The morphological characteristics of the stems, leaves, flowers, sepals and pedicels were assessed in terms of their shape, size, colour, surface features, and the degree and nature of pubescence [4,5].

Quantitative morphometric analysis involved determining the length of the generative shoot, the length and width of the leaflets, the diameter of the flower, and the dimensions of the corolla and sepals. Ten independent plant samples were examined from each of the three collection sites. For quantitative morphometric analysis, images were calibrated using a stage micrometer (10 µm divisions) in ImageJ software (NIH, Bethesda, MD, USA; version 1.54). Measurements were performed using the Measure function, with 10 independent measurements obtained for each parameter per sample.

### 4.3. Microscopical Analysis

Prior to sectioning, the dried above-ground organs were softened in a 1:1:1 (*v*/*v*/*v*) mixture of glycerol, 96% ethanol, and purified water [4,5,31]. Transverse sections of leaves, stems, corollas, sepals, and pedicels were prepared manually and using a MEV cryostat (SLEE Medical GmbH, Mainz, Germany; −35 °C) and a ROTMIK-1 rotary microtome (Orion Medic, Saint Petersburg, Russia). Stem and pedicel sections were obtained from the middle portion of generative shoots, while leaf sections were taken from the central part of apical leaflets. The sections were mounted in glycerol and covered with coverslips.

Microscopical examination was carried out using a Biolab binocular optical microscope (Guangzhou MeCan Medical Limited, Guangzhou, Guangdong, China) and an EX30 vertical laboratory microscope (Ningbo Sunny Instruments Co., Ltd., Ningbo, China) equipped with a 3.1 MP digital camera (Ningbo Sunny Instruments Co., Ltd., Ningbo, China). Observations were performed at magnification combinations of 16 × 4, 16 × 10, and 16 × 45.

The structure of the epidermis, type and dimensions of the stomatal apparatus, shape of epidermal cells, trichome characteristics, mesophyll thickness, organisation of the vascular system, arrangement of mechanical tissues, and secretory structures were assessed. For each anatomical structure, 10 measurements were performed on samples from each collection site.

Anatomical descriptions were compiled in accordance with generally accepted botanical–anatomical principles and the requirements of the State Pharmacopoeia of the Republic of Kazakhstan, the Pharmacopoeia of the Eurasian Economic Union, and international pharmacopoeias [32,33,34,35,36,37].

### 4.4. Histochemical Analysis

For histochemical investigation, dried samples were preliminarily softened in a mixture of 70% ethanol, glycerol, and purified water in a ratio of 1:1:1 [6,38]. Reactions were performed on transverse sections of stems, leaves, corollas, and pedicels. To detect individual groups of primary and secondary metabolites, specific qualitative reactions for phenolic compounds, flavonoids, tannins, polysaccharides, starch, alkaloids, and essential oils were employed. Reagents were obtained from Sigma-Aldrich (St. Louis, MO, USA) and Centralchem (Bratislava, Slovakia).

The presence of the respective group of compounds was assessed by the appearance of characteristic colouration and its localisation in individual tissues and cellular structures. Negative controls were performed on parallel sections without the addition of diagnostic reagent. Microphotographs were obtained under identical lighting conditions and processed using Paint 10.1 software.

### 4.5. Physicochemical Analysis

Quality parameters of the plant raw material, including moisture content, total ash, content of extraneous impurities, and extractive substances, were determined by pharmacopoeial methods in accordance with the requirements of the Pharmacopoeia of the Eurasian Economic Union, the European Pharmacopoeia 11.0, and applicable GOST standards [36,37,38,39,40,41,42,43,44,45,46,47,48,49,50,51]. The content of major groups of biologically active substances, including total polyphenols, flavonoids, tannins, β-carotene, α-tocopherol, and antioxidant components, was determined by spectrophotometric, titrimetric, and chromatographic methods. All determinations were performed in at least three replicates.

### 4.6. Preparation of Dasiphora parvifolia Extracts

The dried above-ground of *Dasiphora parvifolia* were ground to a particle size of 3–5 mm and sieved through a sieve with a mesh diameter of 5 mm. For each extraction variant, 10.0 g of air-dried plant raw material was used. 70% and 90% ethanol, as well as purified water, were employed as extractants. Extracts were obtained by percolation and ultrasonic-assisted extraction. The volume of extractant was calculated taking into account the raw material-to-extractant ratio and the absorption coefficient of the plant material. The main parameters of raw material preparation are presented in Table 11, and the experimental conditions for extract production are given in Table 12.

#### 4.6.1. Percolation

For percolation, 10.0 g of the powdered plant material was moistened with the corresponding hydroethanolic extractant and kept in a closed vessel for 4 h to ensure uniform swelling. The material was subsequently transferred into a percolator without excessive compaction. Extraction was performed with 70% or 90% ethanol at room temperature for 48 h.

The resulting extracts were filtered and concentrated under reduced pressure at a temperature not exceeding 18–25 °C. The percolation extracts were designated DPPE70 and DPPE90.

#### 4.6.2. Ultrasound-Assisted Extraction

Ultrasound-assisted extraction was performed at 20–22 °C using an ultrasonic bath operating at 120 W and 40 kHz. The total sonication time was 1.5 h and consisted of three consecutive 30-min cycles. The temperature of the extraction mixture was controlled between cycles to prevent excessive heating.

The influence of extractant composition, plant material-to-extractant ratio and preliminary soaking duration was evaluated. After extraction, the samples were filtered and concentrated at a temperature not exceeding 25 °C. The extraction conditions and corresponding extract codes are summarized in Table 12.

#### 4.6.3. Determination of Moisture Content of Thick Extracts

The moisture content of the thick extracts obtained after concentration was determined gravimetrically by drying 0.5 g of each extract in a drying oven at 105 °C to constant weight, in accordance with the pharmacopoeial method 2.1.8.16 “Loss on drying of extracts” [36,37]. The average moisture content of the three thick extracts (DPPE70, DPUE70-24, and DPUEW5) was 22.0 ± 1.5%.

### 4.7. HPLC-UV and HPLC-ESI-MS Analysis

The qualitative and quantitative profiling of phenolic compounds was performed by high-performance liquid chromatography with UV detection and additional confirmation by electrospray ionization mass spectrometry. The instrumental and analytical conditions are summarized in Table 13.

Compound identification was performed by comparison of retention times with authentic reference standards and confirmed by ESI-MS in full-scan mode.

The method was used for comparative quantification of phenolic compounds under strictly identical conditions. However, a formal validation of the quantitative method (including linearity, LOD, LOQ, precision, accuracy, system suitability, and solution stability) was not performed.

The results were expressed as percentages relative to the mass of the dry extract.

The total content of quantitatively determined phenolic compounds was calculated as the sum of gallic acid, chlorogenic acid, caffeic acid, epicatechin, catechin, rutin, hyperoside, quercetin, quercetin-3-glucoside, cynaroside and luteolin.

### 4.8. Primary Antibacterial Screening by the Disc-Diffusion Method

The antibacterial activity of the obtained extracts was evaluated by the disc-diffusion method as a primary screening procedure against Gram-positive and Gram-negative bacteria. The principal experimental conditions are summarized in Table 14.

Fresh bacterial cultures were suspended in sterile isotonic sodium chloride solution and adjusted to a turbidity equivalent to 0.5 McFarland.

The standardized bacterial inoculum was uniformly distributed over the entire surface of Mueller–Hinton agar. For the antifungal assay, a fresh culture of Candida albicans ATCC 10231 was prepared and inoculated onto Sabouraud Dextrose Agar (SDA).

The disc-diffusion method was used for comparative primary screening. Extracts producing the largest and most reproducible inhibition zones were selected for subsequent quantitative determination of the minimum inhibitory concentration (MIC) and minimum bactericidal concentration (MBC) against bacterial strains and the minimum fungicidal concentration (MFC) against *C. albicans*.

### 4.9. Statistical Analysis of Extracts and Antibacterial Activity

The analytical and antibacterial determinations were performed in at least three independent replicates, and the results are expressed as mean ± standard deviation (SD).

Extraction conditions were compared based on the total content of quantitatively determined phenolic compounds.

When the assumptions of normality and homogeneity of variance were met, one-way analysis of variance (ANOVA) was performed. Statistical significance was set at *p* ≤ 0.05. ANOVA results are reported as F-statistics, degrees of freedom, and exact *p*-values.

## 5. Conclusions

In the present study, a comprehensive pharmacognostic evaluation of *Dasiphora parvifolia* growing in Central Kazakhstan has been performed encompassing morphological, anatomical, histochemical, physicochemical, and phytochemical analysis of the medicinal plant raw material. A complex of diagnostic macro- and microscopic features has been established, ensuring reliable identification of the species and its differentiation from closely related representatives of the genus *Dasiphora*, primarily *D. fruticosa* and *D. phyllocalyx*, which is of considerable importance for subsequent raw material standardisation.

It has been demonstrated that the investigated raw material is characterised by a high content of phenolic compounds, including total polyphenols (3.70%), tannins (2.67%), and flavonoids (1.36%), and also contains significant amounts of lipophilic antioxidants, particularly β-carotene (330.08 mg/kg) and α-tocopherol (138.0 mg%). Chromatographic analysis confirmed the rich phenolic profile of the extracts, with dominance of gallic acid (up to 3.61%) and chlorogenic acid (up to 2.94%), hyperoside (1.82%), and cynaroside (2.28%). Comparative evaluation of extraction methods demonstrated that ultrasonic-assisted extraction with 70% ethanol and 24-h pre-maceration (DPUE70-24) provides 21.53 mg/g of phenols, which constitutes 73% of the percolation yield (29.59 mg/g), with a total processing time of approximately 25.5 h (including 24 h pre-maceration) compared with 48 h for percolation, and an active sonication time of only 1.5 h. Preliminary antimicrobial screening revealed maximum activity of the DPUE70-24 extract against *Staphylococcus aureus* (inhibition zone 22.0 mm), whereas DPPE70 was most active against *Pseudomonas aeruginosa* (17.0 mm).

The totality of the obtained results confirms the potential of *D. parvifolia* as a novel source of medicinal plant raw material and provides a preliminary scientific basis that may guide the future development of standardization methods, further investigation of pharmacological activity.

## Figures and Tables

**Table 1:** Morphometric characteristics of the above-ground organs of *Dasiphora parvifolia*.

Morphological Object	Morphometric Parameter	*n*	Mean ± SD	Min–Max	F(2, 27)	*p*-Value
Compound leaf	Length, mm	10	9.31 ± 1.70	6.0–12.0	0.027	0.973
	Width, mm	10	4.89 ± 0.93	3.0–6.5	0.012	0.988
	Number of leaflets	10	5.57 ± 0.77	4–7	0.059	0.943
Individual leaflet	Length, mm	10	6.91 ± 0.64	6.0–8.0	0.223	0.802
	Width, mm	10	1.14 ± 0.34	0.6–2.0	0.053	0.948
Petiole	Length, mm	10	5.05 ± 1.02	3.0–7.0	0.068	0.934
Stem	Length, cm	10	17.39 ± 5.64	10.2–27.5	0.112	0.894
	Diameter, mm	10	0.19 ± 0.060	0.10–0.30	0.029	0.972
Flower	Diameter, mm	10	6.92 ± 1.84	4.0–11.3	0.047	0.954
Corolla	Length, mm	10	6.05 ± 1.13	4.0–8.0	0.130	0.879
Sepal	Width, mm	10	5.41 ± 1.17	3.5–8.0	0.018	0.982
	Length, mm	10	5.09 ± 0.36	4.5–6.0	0.330	0.722

Note: Data from 10 plants per site (three sites, total *n* = 30) are pooled, as no significant inter-site differences were observed. F(2, 27) = 0.012–0.330, *p* > 0.05 for all comparisons.

**Table 2:** Comparative morphological study of three species of the genus *Dasiphora*.

Diagnostic Feature	*Dasiphora fruticosa* [9,11]	*Dasiphora parvifolia*	*Dasiphora phyllocalyx* [11,25]
Life form	Branched shrub 20–150 cm tall	Low-growing subshrub 15–80 cm tall	Low-growing subshrub 5–30 cm tall
Stem and bark	Stems erect; bark reddish-brown or grey, exfoliating; young shoots densely covered with silky hairs	Stems erect or ascending, strongly branched; bark greyish-brown, partially exfoliating; young shoots with silky or slightly tomentose pubescence	Stems predominantly erect; generative shoots often purplish
Leaf shape and pubescence	Pinnately compound leaves, usually of 5 leaflets; leaflets oblong, ovate or lanceolate, with entire margins and appressed pubescence; lower surface whitish silky	Leaves mostly of 4–7 linear or linear-lanceolate leaflets with acute apices and slightly revolute margins; upper surface greyish-green, lower whitish, densely covered with silky hairs	Leaves mostly of 5 elliptic or linear-lanceolate leaflets with slightly revolute margins and sparse or moderate soft pubescence
Flower size and arrangement	Flowers solitary, axillary or in loose terminal inflorescences; diameter up to 25 mm	Flowers solitary or in small loose inflorescences; relatively small, mean diameter 6.93 ± 2.20 mm	Flowers solitary, relatively large compared to plant size; diameter up to 30 mm
Hypanthium	Covered with dense appressed or silky hairs	Light green, covered with appressed silky hairs	Densely covered with long white hairs
Outer and inner sepals	Outer sepals linear-lanceolate, equal to or slightly exceeding inner ones; inner sepals ovate, acute	Outer sepals broadly ovate or linear-lanceolate, with obtuse or rounded apices; inner ones smaller, lanceolate and acute	Outer sepals leaf-like, often bifid or divided into 2–3 distinct acute lobes

**Table 3:** Histochemical analysis of the above-ground organs of *Dasiphora parvifolia*.

Compound Group	Reagent and Diagnostic Colouration	Leaf	Corolla	Stem	Pedicel
Flavonoids and other phenolic compounds	1% alcoholic solution of FeCl_3_; dark blue or greenish-black colouration	Epidermis, mesophyll and vascular tissues	Predominantly epidermis	Bark, cortical parenchyma, xylem vessels and pith parenchyma	Cortical parenchyma and vascular bundles
Tannins and other phenolic compounds	10% alcoholic solution of K_2_Cr_2_O_7_; brown or yellowish-brown colouration	Mesophyll and vascular tissues; weaker in epidermis	Predominantly veins and vascular bundles	Bark and xylem tissues	Vascular bundles and pith parenchyma
Alkaloids	Dragendorff’s reagent; brown or dark colouration	Trace amounts in mesophyll	Along vascular bundles	Bark and vascular region	Vascular bundles
Essential oils	Methylene blue; blue colouration	Not detected	Not detected	Not detected	Not detected
Sesquiterpene lactones	Vanillin and concentrated H_2_SO_4_; yellow colouration	Not detected	Not detected	Not detected	Not detected
Polysaccharides	10% thymol solution and concentrated H_2_SO_4_; orange-red colouration	Not detected	Not detected	Not detected	Not detected
Starch	Lugol’s solution; blue colouration	Not detected	Not detected	Not detected	Not detected

**Table 4:** Quality parameters and extractive substance content in the above-ground parts of *Dasiphora parvifolia*.

Parameter	Value
Loss on drying, %	2.61 ± 0.02
Total ash, %	5.55 ± 0.04
Ash insoluble in 10% HCl, %	0.46 ± 0.07
Extraneous impurities, %	0.21 ± 0.007
Extractive substances, water, %	36.09 ± 0.24
Extractive substances, 30% ethanol, %	37.61 ± 0.20
Extractive substances, 50% ethanol, %	39.86 ± 0.30
Extractive substances, 70% ethanol, %	42.36 ± 0.50
Extractive substances, 90% ethanol, %	31.32 ± 0.21

**Table 5:** Content of components of the hydrophilic antioxidant complex in the above-ground parts of *D. parvifolia* (M ± SD, calculated on absolutely dry raw material basis).

Fraction	Parameter	Value	Analytical Method
Hydrophilic	Total polyphenols, mg-equiv gallic acid/g	3.70 ± 0.04	Spectrophotometry
Hydrophilic	Total flavonoids, mg-equiv rutin/g	1.36 ± 0.01	Spectrophotometry
Hydrophilic	Tannins, %	2.67 ± 0.03	Titrimetry
Hydrophilic	Ascorbic acid, mg%	11.2 ± 0.10	Titrimetry
Hydrophilic	Water-soluble antioxidants, mg-equiv quercetin/g	1.81 ± 0.0096	Amperometry
Lipophilic	β-Carotene, mg/kg	330.08 ± 2.65	Spectrophotometry
Lipophilic	α-Tocopherol, mg%	138.0 ± 0.8	HPLC
Lipophilic	Fat-soluble antioxidants, mg-equiv quercetin/g	0.87 ± 0.0014	Amperometry

Note: Results are expressed on absolutely dry raw material basis as M ± SD; for the lipophilic fraction, *n* = 2.

**Table 6:** Conditions for preparation of *Dasiphora parvifolia* extracts.

Extract Code	Method	Extractant	Raw Material:Extractant Ratio	Specific Conditions
DPPE70	Percolation	70% EtOH	1:10	Classical extraction
DPPE90	Percolation	90% EtOH	1:10	Classical extraction
DPUE705	Ultrasound	70% EtOH	1:5	Without pre-maceration
DPUE7010	Ultrasound	70% EtOH	1:10	Without pre-maceration
DPUE7020	Ultrasound	70% EtOH	1:20	Without pre-maceration
DPUE90	Ultrasound	90% EtOH	1:20	Without pre-maceration
DPUEW5	Ultrasound	Water	1:5	Without pre-maceration
DPUEW10	Ultrasound	Water	1:10	Without pre-maceration
DPUE70-24	Ultrasound	70% EtOH	1:20	Pre-maceration for 24 h
DPUE90-24	Ultrasound	90% EtOH	1:20	Pre-maceration for 24 h

**Table 7:** HPLC conditions used for phytochemical profiling.

Parameter	Value
Column	Zorbax Eclipse Plus C18 (150 mm × 4.6 mm, 3.5 μm)
Temperature	30 °C
Flow rate	0.4 mL/min
Injection volume	20 μL
Mobile phase A	0.1% HCOOH in water
Mobile phase B	Acetonitrile
Detection	280 and 360 nm
Compound confirmation	HPLC-ESI/MS

**Table 8:** Phenolic composition of *Dasiphora parvifolia* extracts determined by HPLC.

Extract	Hydroxybenzoic Acids	Hydroxycinnamic Acids		Flavan-3-ols	Flavonols				Flavones		Total Identified Phenolics (%)
	Gallic Acid	Chlorogenic Acid	Caffeic Acid	Catechin	Rutin	Hyperoside	Quercetin	Quercetin-3-Glucoside	Cynaroside	Luteolin	
Detection wavelength (nm)	280	280	280	360	360	360	360	360	360	360	
DPPE70	0.99	2.01	–	0.16	0.34	1.82	0.07	-	2.28	0.01	7.68
DPPE90	0.39	1.77	–	0.17	0.31	0.60	0.03	-	0.79	0.004	4.06
DPUE70-5	2.90	0.19	0.04	0.02	0.27	0.41	0.008	-	-	-	3.84
DPUE70-10	1.81	0.36	0.007	0.029	0.09	0.43	0.028	0.62	1.82	-	5.19
DPUE70 (1:20)	0.65	2.52	–	–	0.37	1.21	0.05	-	0.05	0.008	4.86
DPUE90	0.30	1.19	–	–	0.16	0.25	0.02	-	0.42	0.01	2.35
DPUEW5	3.61	0.87	0.021	0.063	0.18	0.83	0.062	1.17	-	-	6.81
DPUEW10	2.77	0.25	0.028	0.029	0.14	0.28	0.009	0.52	-	-	4.03
DPUE70 (24 h)	0.60	2.94	–	–	0.39	1.48	0.05	-	2.06	0.007	7.53
DPUE90 (24 h)	0.14	0.63	–	0.01	–	–	-	-	-	-	0.78

Notes: Abbreviations: DPPE—percolation extract; DPUE—ultrasound-assisted extract; W—purified water. Values are expressed as % of extract dry weight. Compound identification was performed by HPLC-UV and HPLC-ESI-MS, with *m*/*z* confirmation using reference standards. Detection wavelengths were 280 nm for phenolic acids and 360 nm for flavan-3-ols, flavonols, and flavones. “–” indicates not detected.

**Table 9:** Comparison of extraction efficiency for selected *Dasiphora parvifolia* extracts.

Extract	Mass of Wet Thick Extract (g)	Moisture of Thick Extract (%)	Average Moisture Content of the Thick Extracts	Dry Extract Yield (g per 10 g Raw Material)	Total Phenolics, % of Dry Extract	Total Phenolics (mg/g Raw Material)
DPPE70	4.870	22.0	22.0 ± 1.5%	3.7402	7.68	28.72
DPUE70-24	3.670	23.5		2.7635	7.53	20.81
DPUEW5	0.684	20.5		0.53352	6.81	3.63

**Table 10:** Preliminary antimicrobial activity of *Dasiphora parvifolia* extracts.

Test Microorganism	DPPE70	DPUE70-24	Positive Control	70% EtOH
*Bacillus subtilis* ATCC 6633	6.0 ± 0.2	8.0 ± 0.9	Chloramphenicol 15.0 ± 0.2	6
*Staphylococcus aureus* ATCC 6538	13.0 ± 0.5	22.0 ± 0.78	Cefoxitin 27.0 ± 1.52	6
*Pseudomonas aeruginosa* ATCC 9027	17.0 ± 0.4	15.0 ± 0.1	Ampicillin 28.0 ± 0.48	6
*Escherichia coli* ATCC 8739	6.0 ± 0.06	6.0 ± 0.3	Ampicillin 28.0 ± 0.53	6
*Candida albicans* ATCC 10231	6.0 ± 0.1	6.0 ± 0.5	Nystatin 12.0 ± 0.2	6

Note: Disc diameter = 6 mm; values are mean ± SD from three independent replicates.

**Table 11:** General parameters of the plant material and extraction procedures.

Parameter	Percolation	Ultrasound-Assisted Extraction
Plant material	above-ground of *D. parvifolia*	above-ground of *D. parvifolia*
Mass of plant material	10.0 g	10.0 g
Material condition	Air-dried	Air-dried
Particle size	3–5 mm	3–5 mm
Sieve aperture	5 mm	5 mm
Extractants	70% and 90% ethanol	70% and 90% ethanol; purified water
Plant material-to-extractant ratio	1:10	1:5, 1:10 and 1:20
Absorption coefficient	3.7 mL/g	3.7 mL/g for hydroethanolic extractants; 2.2 mL/g for water
Preliminary soaking	Yes	Yes
Preliminary soaking time	4 h	Standard soaking or 24 h, depending on the experiment
Extraction temperature	Room temperature	20–22 °C
Total extraction time	48 h	1.5 h (UAE), 25.5 h (UAE-24)
Number of ultrasonic cycles	Not applicable	3
Duration of one ultrasonic cycle	Not applicable	30 min
Ultrasound power	Not applicable	120 W
Ultrasound frequency	Not applicable	40 kHz

**Table 12:** Experimental conditions used for preparation of *D. parvifolia* extracts.

Code	Method	Extractant	Ratio (*w*/*v*)	Solvent Absorption (mg/g)	Initial Volume (mL)	Total Volume (mL)	Soaking	Remarks
DPPE70	Percolation	70% EtOH	1:10	3.7	137	500	4 h	—
DPPE90	Percolation	90% EtOH	1:10	3.7	137	582	4 h	—
DPUE705	UAE	70% EtOH	1:5	3.7	87	261	—	Standard UAE
DPUE7010	UAE	70% EtOH	1:10	3.7	137	411	—	Standard UAE
DPUE7020	UAE	70% EtOH	1:20	3.7	237	711	—	Standard UAE
DPUE90	UAE	90% EtOH	1:20	3.7	237	711	—	Standard UAE
DPUEW5	UAE	Purified water	1:5	2.2	72	216	—	Standard UAE
DPUEW10	UAE	Purified water	1:10	2.2	122	366	—	Standard UAE
DPUE70-24	UAE	70% EtOH	1:20	3.7	237	711	24 h	Pre-soaked
DPUE90-24	UAE	90% EtOH	1:20	3.7	237	711	24 h	Pre-soaked

Abbreviations: EtOH—ethanol. Note: The “Initial Volume” refers to the volume of solvent used in a single extraction cycle, calculated based on the absorption coefficient of the plant material. The “Total volume” represents the cumulative solvent volume used throughout the extraction process, including three cycles for UAE and continuous solvent consumption during 48 h of percolation.

**Table 13:** Instrumental conditions for HPLC-UV and HPLC-ESI-MS analysis.

Parameter	Analytical Conditions
Analytical methods	HPLC-UV and HPLC-ESI-MS
Main objective	Identification and quantitative determination of phenolic compounds
HPLC system	Agilent 1260 Infinity (Agilent Technologies, Inc., Santa Clara, CA, USA)
Pump	G1311C 1260 Pump VL, quaternary (Agilent Technologies, Inc., Santa Clara, CA, USA)
Autosampler	G1329B 1260 ALS (Agilent Technologies, Inc., Santa Clara, CA, USA)
Column thermostat	G1316A 1260 TCC (Agilent Technologies, Inc., Santa Clara, CA, USA)
UV detector	G1314C 1260 VWD VL+ (Agilent Technologies, Inc., Santa Clara, CA, USA)
Mass spectrometer	G6130A quadrupole LC-MS (Agilent Technologies, Inc., Santa Clara, CA, USA)
Ionization mode	Electrospray ionization
Software	ChemStation with Windows NT (Agilent Technologies, Inc., Santa Clara, CA, USA)
Mobile phase components	Acetonitrile, formic acid and Milli-Q water
Acetonitrile	≥99.9% (Sigma-Aldrich, Saint-Quentin-Fallavier, France)
Formic acid	99–100% (VWR Chemicals, Rosny-sous-Bois, France)
Water	Milli-Q (Millipore, Molsheim, France)
Number of reference standards	20
Primary identification	Retention time compared with authentic standards
Additional confirmation	ESI-MS mass spectra
Quantitative approach	External-standard method
Detection wavelengths	280 and 360 nm
Column	Zorbax Eclipse Plus C18 (150 mm × 4.6 mm, 3.5 μm)
Column temperature	30 °C
Flow rate	0.4 mL/min
Injection volume	20 µL
Gradient program	0-5-10-15-45-50-55-60 min: 3-10-20-30-40-30-20-3% B

**Table 14:** Conditions of the primary disc-diffusion antibacterial screening.

Parameter	Experimental Conditions
Screening method	Disc-diffusion method
Test microorganisms	Gram-positive and Gram-negative bacteria
Gram-positive strains	*Bacillus subtilis* ATCC 6633; *Staphylococcus aureus* ATCC 6538
Gram-negative strains	*Pseudomonas aeruginosa* ATCC 9027; *Escherichia coli* ATCC 8739
Culture medium	Mueller–Hinton agar, Sabouraud Dextrose Agar
Inoculum preparation	Fresh 18–24 h bacterial culture
Inoculum standardization	0.5 McFarland
Approximate bacterial concentration	1.5 × 10^8^ CFU/mL
Disc material	Sterile paper discs
Disc diameter	6 mm
Extract concentration	1 mg/mL
Volume applied per disc	10 µL
Extract amount per disc	0.01 mg
Extract solvent	70% ethanol
Negative control	10 µL of 70% ethanol
Positive control for Gram-positive bacteria	Chloramphenicol (30 µg/disc); Cefoxitin (30 µg/disc); Ampicillin (30 µg/disc)
Positive control for Gram-negative bacteria	Ampicillin (30 µg/disc)
Fungal strain	*Candida albicans* ATCC 10231
Positive control for Candida albicans	Nystatin (30 µg/disc)
Pre-diffusion period	not used
Incubation temperature	35–37 °C
Incubation time	18–24 h
Number of replicates	At least three
Outcome measure	Diameter of the complete inhibition zone, mm

## Data Availability

The original contributions presented in this study are included in the article/Supplementary Material. Further inquiries can be directed to the corresponding authors.

## Supplementary Materials

The following supporting information can be downloaded at: https://www.mdpi.com/article/10.3390/ph19091443/s1, Figure S1: Representative HPLC-UV chromatograms of *Dasiphora parvifolia* extracts; Table S1: Summary of one-way ANOVA results for morphometric data (three collection sites, *n* = 10 per site, total *n* = 30); Table S2: Summary of one-way ANOVA results for anatomical data (three collection sites, *n* = 10 per site, total *n* = 30); Table S3: Identified phenolic compounds in DPPE70 extract by HPLC-UV/ESI-MS; Table S4: Identified phenolic compounds in DPUEW5 extract by HPLC-UV/ESI-MS; Table S5: Identified phenolic compounds in DPUE70-24 extract (by HPLC-UV/ESI-MS.
