# Supplementary material for: Dasiphora parvifolia: Pharmacognostic Standardization and Antimicrobial Potential
Source: Pharmaceuticals (Basel). 2026 Sep 11;19(9):1443. doi: 10.3390/ph19091443 (PMC13610915; doi:10.3390/ph19091443)
Supplement: Supplementary file 1 [file pharmaceuticals-19-01443-s001.zip › Supplementary Materials.pdf]

Supplementary Materials

Table S1. Summary of one-way ANOVA results for morphometric data (three collection sites, n = 10 per site, total n = 30)

Parameter	Karkaraly Mountains (n=10) Mean ± SD	Korneyev Forests (n=10) Mean ± SD	Bektauata Mountains. (n=10) Mean ± SD	Overall Mean (n=30) Mean ± SD	Min	Max	df1	df2	SSB	SSW	MSB	MSW	F(2, 27)	p-value
Leaf														
Length (mm)	9.22 ± 1.98	9.30 ± 1.49	9.40 ± 1.71	9.31 ± 1.70	6.0	12.0	2	27	0.1627	81.64	0.0814	3.024	**0.027**	**0.973**
Width (mm)	4.88 ± 1.09	4.87 ± 0.83	4.92 ± 0.97	4.89 ± 0.93	3.0	6.5	2	27	0.0140	29.60	0.0070	1.096	**0.012**	**0.988**
Number of leaflets	5.50 ± 0.85	5.60 ± 0.70	5.60 ± 0.70	5.57 ± 0.77	4	7	2	27	0.0667	15.30	0.0334	0.567	**0.059**	**0.943**
Leaflet														
Length (mm)	6.74 ± 0.69	7.01 ± 0.55	6.99 ± 0.58	6.91 ± 0.64	6.0	8.0	2	27	0.4520	27.40	0.2260	1.015	**0.223**	**0.802**
Width (mm)	1.13 ± 0.40	1.17 ± 0.26	1.12 ± 0.33	1.14 ± 0.34	0.6	2.0	2	27	0.0140	3.58	0.0070	0.133	**0.053**	**0.948**
Petiole														
Length (mm)	4.95 ± 1.10	5.10 ± 0.94	5.11 ± 0.82	5.05 ± 1.02	3.0	7.0	2	27	0.1600	31.94	0.0800	1.183	**0.068**	**0.934**
Stem														
Length (cm)	17.71 ± 5.83	17.45 ± 5.22	17.00 ± 5.86	17.39 ± 5.64	10.2	27.5	2	27	2.5800	312.20	1.2900	11.56	**0.112**	**0.894**
Diameter (cm)	0.186 ± 0.064	0.189 ± 0.055	0.182 ± 0.060	0.186 ± 0.060	0.10	0.30	2	27	0.00025	0.116	0.00012	0.0043	**0.029**	**0.972**
Flower														
Diameter (mm)	6.93 ± 2.20	7.05 ± 1.78	6.78 ± 1.80	6.92 ± 1.84	4.0	11.3	2	27	0.3660	105.24	0.1830	3.898	**0.047**	**0.954**
Corolla														
Length (mm)	6.29 ± 1.22	6.02 ± 0.94	5.83 ± 1.02	6.05 ± 1.13	4.0	8.0	2	27	0.3850	40.12	0.1925	1.486	**0.130**	**0.879**
Sepal														
Width (mm)	5.69 ± 1.32	5.28 ± 0.91	5.25 ± 1.02	5.41 ± 1.17	3.5	8.0	2	27	0.0527	39.80	0.0264	1.474	**0.018**	**0.982**
Length (mm)	5.09 ± 0.42	5.17 ± 0.38	5.02 ± 0.30	5.09 ± 0.36	4.5	6.0	2	27	0.1167	4.78	0.0584	0.177	**0.330**	**0.722**

Note: df1-degrees of freedom between groups; df2-degrees of freedom within groups. SSB-sum of squares between groups; SSW-sum of squares within groups; MSB-mean square between groups; MSW-mean square within groups. All p-values > 0.05, indicating no significant differences between the three collection sites.

Table S2. Summary of one-way ANOVA results for anatomical data (three collection sites, n = 10 per site, total n = 30)

Parameter	df1	df2	F	p-value
Stomata length (μm)	2	27	0.45	0.643
Stomata width (μm)	2	27	0.52	0.601
Stomatal index (%)	2	27	0.68	0.515
Epidermis length (μm)	2	27	0.38	0.688
Epidermis width (μm)	2	27	0.41	0.668
Cell density (per 1 mm²)	2	27	0.62	0.546
Trichome length (μm)	2	27	0.48	0.624
Trichome width (μm)	2	27	0.35	0.709
Trichome diameter (μm)	2	27	0.30	0.744
Trichome density (per 1 mm²)	2	27	0.55	0.584
Mesophyll thickness (μm)	2	27	0.28	0.758
Palisade thickness (μm)	2	27	0.50	0.612
Spongy thickness (μm)	2	27	0.33	0.722
Vascular bundle diameter (μm)	2	27	0.28	0.758

Note: df1-degrees of freedom between groups; df2-degrees of freedom within groups. All p-values > 0.05, indicating no significant differences between the three collection sites.

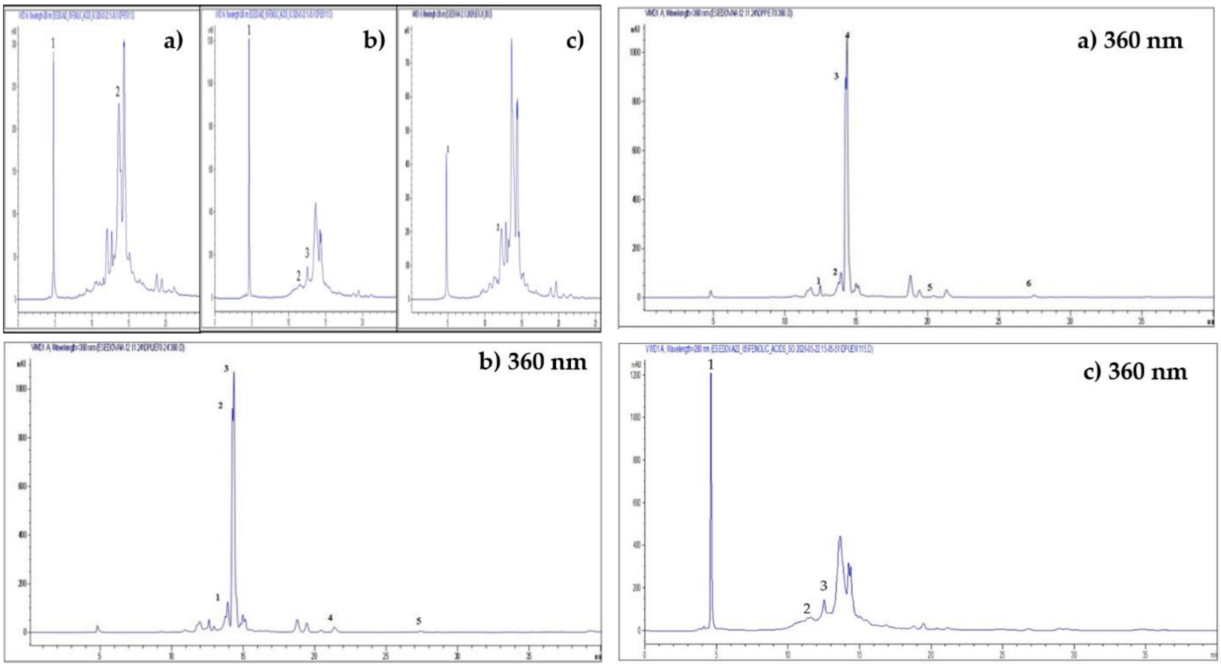

Figure S1. Representative HPLC-UV chromatograms of *Dasiphora parvifolia* extracts: **a)** DPPE70 (percolation, 70% ethanol); **b)** DPUEW5 (ultrasound-assisted extraction, purified water); **c)** DPUE70-24 (ultrasound-assisted extraction, 70% ethanol, 24 h pre-maceration). Left panels: detection at 280 nm (phenolic acids); right panels: detection at 360 nm (flavonoids). Peak assignments: 280 nm 1-gallic acid; 2-chlorogenic acid; 3-caffeic acid (only in DPUEW5). 360 nm 1-

catechin (in **a)** and **b)**); 2-rutin (in **a), b), c)**); 3-hyperoside (in a**), b), c)**); 4-cynaroside (in **a)** and **b)**); 5-quercetin-3-glucoside (only in **c)**); 6-quercetin (in a**), b), c)**); 7-luteolin (in **a)** and **b**).

Table S3. Identified phenolic compounds in DPPE70 extract by HPLC-UV/ESI-MS.

**Peak**	**Retention time (min)**	**[M-H]⁻ (m/z)**	**Compound**	**Class**	**Content (% of dry extract)**	**Detection wavelength (nm)**
**1**	4.787	169	Gallic acid	Phenolic acid	0.99	280
**2**	14.474	353	Chlorogenic acid	Hydroxycinnamic acid	2.01	280
**1**	12.473	289	Catechin	Flavan-3-ol	0.16	360
**2**	13.926	609	Rutin	Flavonol glycoside	0.34	360
**3**	14.254	463	Hyperoside	Flavonol glycoside	1.82	360
**4**	14.352	448	Cynaroside	Flavone glycoside	2.28	360
**5**	21.315	302	Quercetin	Flavonol (aglycone)	0.07	360
**6**	27.459	286	Luteolin	Flavone (aglycone)	0.01	360

Table S4. Identified phenolic compounds in DPUEW5 extract by HPLC-UV/ESI-MS

**Peak**	**Retention time (min)**	**[M-H]⁻ (m/z)**	**Compound**	**Class**	**Content (% of dry extract)**	**Detection wavelength (nm)**
1	4.613	169	Gallic acid	Phenolic acid	3.61	280
2	12.530	179	Caffeic acid	Hydroxycinnamic acid	0.021	280
3	13.652	353	Chlorogenic acid	Hydroxycinnamic acid	0.87	280
1	12.579	289	Catechin	Flavan-3-ol	0.063	360
2	13.683	463	Quercetin-3-glucoside	Flavonol glycoside	1.17	360
3	13.895	609	Rutin	Flavonol glycoside	0.18	360
4	14.282	463	Hyperoside	Flavonol glycoside	0.83	360
5	21.291	302	Quercetin	Flavonol (aglycone)	0.062	36

Table S5. Identified phenolic compounds in DPUE70-24 extract (by HPLC-UV/ESI-MS

**Peak**	**Retention time (min)**	**[M-H]⁻ (m/z)**	**Compound**	**Class**	**Content (% of dry extract)**	**Detection wavelength (nm)**

**1**	4.782	169	Gallic acid	Phenolic acid	0.60	280
**2**	12.834	353	Chlorogenic acid	Hydroxycinnamic acid	2.94	280
**1**	13.916	609	Rutin	Flavonol glycoside	0.39	360
**2**	14.252	463	Hyperoside	Flavonol glycoside	1.48	360
**3**	14.348	448	Cynaroside	Flavone glycoside	2.06	360
**4**	21.378	302	Quercetin	Flavonol (aglycone)	0.05	360
**5**	27.413	286	Luteolin	Flavone (aglycone)	0.007	360
